# Waking Up: Searching for Spirituality without Religion

**DOI:** 10.1192/pb.bp.115.053090

**Published:** 2017-02

**Authors:** Matthew M Nour

Sam Harris has been waiting to write this book for over a decade. This may surprise some. The subject matter – dealing reverently with human spiritual experience – is at odds with Harris' (in)famous public persona as a strident critic of religion. Yet, for the past 20 years Harris, who has degrees in philosophy and neuroscience, has been on a personal quest in search of ‘transformative insights about the nature of one's own consciousness’.

Harris defines spiritual practice as the efforts people make, through meditation, use of psychedelics or other means, to fully bring their minds into the present. This practice leads to the insight that our sense of having a unified self is an illusion and that this illusion causes us much psychological suffering. Harris aims to convince his reader of this using philosophical thought experiments, discoveries of contemporary neuroscience and personal experience. He also encourages his reader to test these hypotheses about human consciousness ‘in the laboratory of your own mind’, through meditation practices inspired by Buddhist Dzogchen and Vipassana teaching. He argues that these spiritual insights can be accepted independently of the metaphysical baggage of traditional religion, and laments that until recently they have been under-investigated by an ‘impoverished’ neuroscience.

The resulting book is an ambitious mosaic: part memoir, part neuropsychology text and part meditation guide. A key strength is Harris' clear, lively and personal writing style, which instils the prose with an endearing conversational air. Many readers will feel, however, that by focusing almost exclusively on solitary meditation practices and psychedelic drug-induced experiences, Harris has omitted important dimensions of human spiritual experience, such as the self-transcendence which may be arrived at when contemplating art or engaging in communal ceremonial practices. Moreover, the occasional barbed criticism of monotheistic religion will deter some readers, but play well to the Harris faithful.

*Waking Up* is a book for the general public and is not intended to have a clinical application. Why, then, is it being discussed in the pages of this journal? My answer is twofold. First, as psychiatrists we are interested in all dimensions of human experience. Consequently, the growing scientific interest in the mystical/spiritual experience and its potential therapeutic implications is of great importance for our specialty. Second, psychiatrists are humans and all humans may benefit from being reminded from time to time that our conventional sense of a unified self sitting some 2 inches behind the eyes is likely to be a pernicious illusion.

